# Supplementary material for: Passivation and characterization of charge defects in ambipolar silicon quantum dots
Source: Sci Rep. 2016 Dec 6;6:38127. doi: 10.1038/srep38127 (PMC5138628; doi:10.1038/srep38127)
Supplement: Supplementary Information [file srep38127-s1.pdf]

Supplementary Information: Passivation and characterization of charge defects in ambipolar silicon quantum dots

Paul C. Spruijtenburg, Sergey V. Amitonov, Filipp Mueller, Wilfred G. van der Wiel & Floris A. Zwanenburg

In this supplementary information we will first consider dewetting effects of Al on the surface of SiO_2 at elevated temperatures. Then we will discuss the characteristics of individual barrier cut-off before and after annealing.

1 Dewetting

Dewetting is the breaking of continuity of an initially continuous film caused by differences in free surface energy at liquid-liquid or solid-liquid interfaces. In our case the interface consists of Al and SiO_2. At elevated temperatures thin films of Al, while still below its melting point, become mobile. Dewetting becomes energetically favourable under the correct circumstances: temperature, graininess of the film, free surface energy at the Al/SiO_2 and vacuum/Al interface.

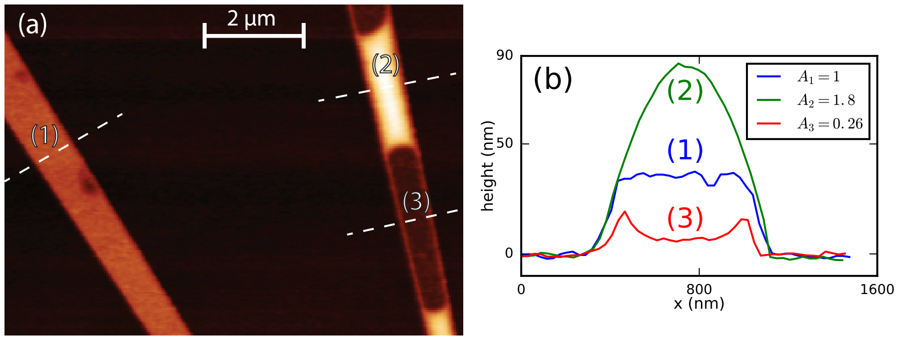

Supplementary Figure S1: Dewetting behaviour of pure aluminum on SiO_2. (a) shows the AFM image overview of a device after 400°C annealing. (b) shows the height at the various linecuts and the areas underneath each curve normalized to A_1, the area under curve (1).

Supplementary Figure S1a shows dewetting behaviour as observed in one of our samples after annealing, without the presence of an Al2O3 overlayer. The left Al electrode, labeled (1), shows partial signs of dewetting starting but still has its as-deposited height of 36 nm. The right electrode shows clear signs of dewetting. At (2) the height has increased from 36 nm as-deposited to a maximum of roughly 90 nm. The material for this height increase has been

moved from (3) to (2). The areas A_i underneath curves taken at (1), (2), and (3) are normalized to A_1. We see that $A_2 + A_3 \simeq 2A_1$, i.e the material "missing" from (3) has moved to (2), and thus the sum equals twice the original. At the interface region between (2) and (3) the convex/concave shape is reminiscent of the classical meniscus in e.g. water, indicating energy minimisation caused by surface tension. This can also be observed on both sides of the electrode at (3). [1]

2 Resonances in individual barriers before and after annealing

Here we will have a look at the individual barriers and their behaviour near their cut-off voltages both before and after annealing. Numbering of the barriers in this section does not necessarily correspond to the numbering used in the main manuscript.

After annealing we observe that the absolute threshold voltages $|V_{\mathrm{Th}}|$ tend to be much lower. The respective cut-off voltages therefore also trend to lower values. For a qualitative comparison we have shifted all curves to $V = 0$ mV when a certain threshold on I_{SD} is reached.

Supplementary Figure S2a shows current-voltage type curves for device A for both $V_{\mathrm{SD}} = 1$ mV and $V_{\mathrm{SD}} = 6.8$ mV. This allows to observe the behaviour of resonances and charge states up to higher charging energies. Before annealing, resonances are present even for $V_{\mathrm{SD}} = 6.8$ mV and dominate the conduction. After annealing (green) the resonances for both bias conditions have significantly decreased.

We have tried to quantify the oscillatory behaviour by using root-mean-square, peak-detection, and histogram analysis. However due to the chaotic behaviour, we have concluded that the trend gleaned from these methods is less clear than from a simple inspection by eye of curves as in Supplementary Figure S2 and S3.

We have also measured another sample of type A, device A2, for which the results are shown in Supplementary Figure S3. This sample shows qualitatively the same results as sample A. Before annealing irregular resonances in the individual current-voltage graphs are often seen. After annealing the resonances are greatly reduced.

On this background of reduced resonances, some resonances are retained. This can be seen for B1 and B5 in Supplementary Figure S3, where a pronounced resonance just below the cut-off voltage is preserved after the annealing process.

References

[1] Thompson, C. V. Solid-State Dewetting of Thin Films. *Annual Review of Materials Research* **42**, 399–434 (2012).

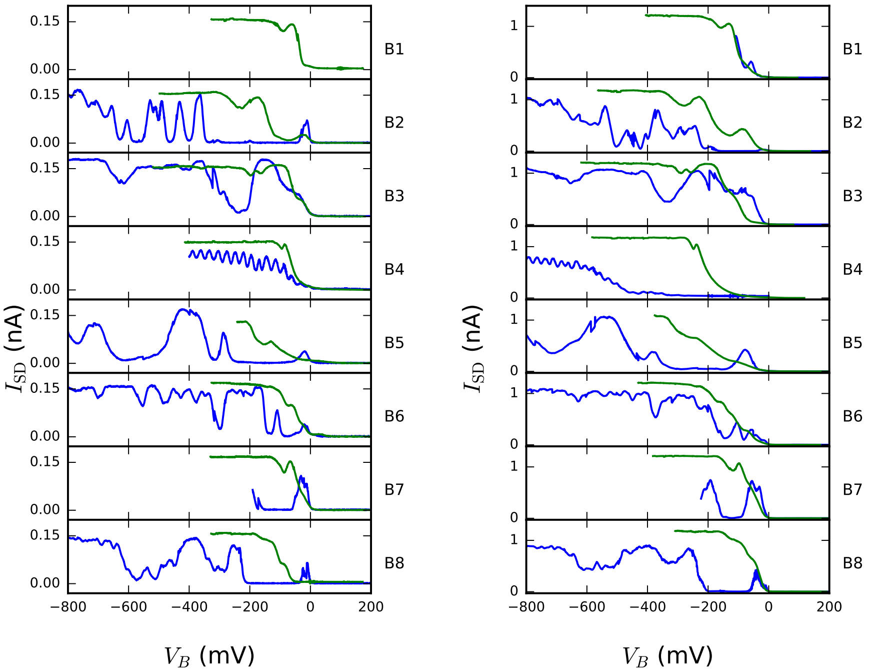

Supplementary Figure S2: Current versus Barrier-gate voltage for all working barriers for device A, before (blue) and after (green) annealing. For (a) $V_{SD} = 1$ mV and (b) $V_{SD} = 6.8$ mV

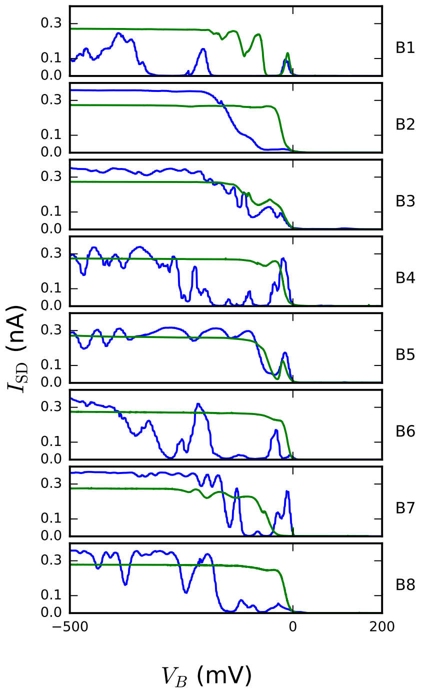

Supplementary Figure S3: Current versus barrier-gate voltage for all working barriers of device A2 before (blue) and after (green) annealing. For each measurement all other barriers are kept transparent (i.e. $|V_B| \gg V_{B\text{Threshold}}$). $V_{SD} = 1.0$ mV
